# Optimization of water and fertilizer management on the intercropping system between maize and mung bean to improve photosynthetic characteristics & water use and to increase plant yield

**DOI:** 10.3389/fpls.2025.1597198

**Published:** 2025-07-21

**Authors:** Mengfan Gao, Xinxin Wang, Renqiang Chen, Huiyan Gao, Xiaoling Wang, Hongquan Liu

**Affiliations:** ^1^ College of Urban and Rural Construction, Hebei Agricultural University, Baoding, China; ^2^ Agricultural Technology Innovation Center in Mountainous Areas of Hebei Province, Hebei Agricultural University, Baoding, China; ^3^ Agricultural Engineering Technology Research Center, National North Mountainous Area, Baoding, China; ^4^ Hebei Mountain Research Institute, Hebei Agricultural University, Baoding, China; ^5^ College of Horticulture, Hebei Agricultural University, Baoding, China; ^6^ North China Water saving Agriculture Key Laboratory, Ministry of Agriculture and Rural Affairs, Baoding, China; ^7^ State Key Laboratory of North China Crop Improvement and Regulation, Hebei Agricultural University, Baoding, China

**Keywords:** intercropping, organic fertilizer, photosynthetic traits, water use efficiency, irrigation

## Abstract

Intercropping has the advantages of improving the canopy environment and enhancing the productivity of farmland. However, the responses of photosynthetic physiological characteristics, canopy structure and yield to different water and fertilizer measures need to be further clarified. The study took the maize/mung bean intercropping as the cultivation mode, used organic fertilizer instead of chemical fertilizer, combined with the upper and lower limits of field water holding capacity to control the irrigation amount, and set up a two-factor field experiment. The research results show that the leaf area index and chlorophyll relative content of maize and mung beans increase first and then decrease with the emergence time of maize and mung beans. Compared with the treatment without organic fertilizer, the leaf area index of maize and mung beans increased by 5.99% - 36.70% and 27.43% - 28.72% respectively, and the chlorophyll relative content increased by 2.31% - 3.47% and 4.59% - 4.63% respectively. Compared with I_0_, leaf area index increased by 9.73% - 33.42% and 6.60% - 17.39% respectively, and chlorophyll relative content increased by 2.75% - 12.68% and 4.14% - 9.12% respectively. The gas exchange parameters and absorbance(Ab) of maize and mung beans showed a trend of increasing first and then decreasing with the emergence time. The net photosynthetic rates of maize and mung beans increased by 5.04% - 47.12% and 11.29% - 26.60% respectively. Maize Pn was strongly positively correlated with chlorophyll relative content and Ab (*R* ^2^>0.5). The three-dimensional growth curves of Pn along with chlorophyll relative content and Ab were S-shaped. As the growth period progressed, the leaves would age, and the gradual decrease of chlorophyll relative content and Ab led to a gradual decrease in maize Pn. Within a certain range, with the increase of irrigation volume, the water use efficiency(WUE) of crops shows a trend of increasing first and then decreasing. Organic fertilizer can significantly improve the WUE of maize/mung beans intercropping crops. In conclusion, optimizing the combination of organic fertilizers and irrigation practices is a win-win strategy that can enhance both grain output and quality.

## 1 Introduction

Maize (*Zea mays* L.), as a key staple crop, faces a rapidly widening gap between production and demand (Wu et al., 2024; Yang et al., 2024). Mung bean (*Vigna radiata* L.), as an economical crop with high nutritional content, exhibits stable market demand. However, the industry faces challenges of reduced yields due to decreasing planting areas and inadequate technical support (Huang et al., 2024). Intercropping of gramineous and legumes represents a typical intercropping system. By leveraging differences in biological characteristics, a scientifically designed composite spatial layout can be established to enhance light availability for crops and create an environment conducive to the harmonious coexistence of both crops in terms of light, nutrients, and water resources (Pelzer et al., 2012; Li et al., 2023; Yang et al., 2023a). Numerous qualitative studies (Bedoussac and Justes, 2011; Chimonyo et al., 2016; Kermah et al., 2017) and systematic reviews (Bedoussac et al., 2015; Du et al., 2018; Tilman, 2020) have demonstrated through practical applications worldwide that intercropping enhances nitrogen use efficiency, resource utilization efficiency, and soil ecosystem capacity by addressing factors such as growth physiology, interspecific competition, and agronomic practices. These findings suggest that intercropping serves as a viable approach to achieving sustainable intensive agricultural production.

Irrigation and nitrogen fertilization are critical factors in agricultural production, directly influencing crop growth and development (Liu et al., 2022; Ma et al., 2022). Water plays a pivotal role in enhancing fertilizer availability, while fertilizers are critical for unlocking the productivity of soil-water systems (Himmelstein et al., 2017). Because of the lack of scientific and efficient irrigation and fertilizer management among local farmers, the amounts of water and fertilizers applied in intercropping systems often exceed the actual crop demands, leading to resource waste and reduced efficiency (Zhang et al., 2011; Chen et al., 2023b). Organic fertilizers, as natural nutrient sources, not only supply multiple essential elements for crop growth but also improve photosynthetic characteristics, thereby enhancing product quality (Jannoura et al., 2014; Adetunji et al., 2020). To mitigate water scarcity in arid and semi-arid regions, understanding water-fertilizer interactions and their underlying physiological mechanisms is fundamental for optimizing water-saving irrigation systems (Xue et al., 2016). Studies demonstrate that appropriate water and fertilizer management helps maintain higher chlorophyll content, delays leaf senescence, and consequently enhances photosynthetic efficiency through sustaining stomatal openness, increasing transpiration rates, and promoting the transport and accumulation of photosynthetic products (Tian et al., 2019; Yang et al., 2023b). For instance, Wu et al. (2023) optimized water-nitrogen management to increase leaf number, improve canopy light conditions, and enhance cotton yield. Similarly, Luo et al. (2021) improved light environments and boosted biomass and yield in intercropped wheat through refined nitrogen application strategies.

Although irrigation and organic fertilizers are widely applied in agricultural practices, current research on optimizing water-fertilizer management to improve plant growth, physiological traits, light energy utilization, biomass, and yield responses in maize/mung bean intercropping systems remains limited. Existing studies predominantly focus on monoculture systems, failing to provide theoretical foundations for agricultural production under maize/mung bean intercropping (Yang et al., 2014; Liu et al., 2017; Luo et al., 2024). Furthermore, strategies to rationally design irrigation schedules (e.g., water volume and timing) based on crop water requirements, enhance coordination between crop water demand and supply, and maximize the systemic advantages of cereal-legume intercropping require further in-depth exploration.

Therefore, this study adopts a maize/mung bean intercropping system as the experimental model, with two independent variables: organic fertilizer application rate and irrigation volume. The objectives are to: 1) investigate the effects of varying organic fertilizer application rates and irrigation volumes on the yield of maize and mung beans; 2) explore the synergistic effects of water-fertilizer interactions on crop photosynthetic and physiological traits; 3) examine the regulatory roles of organic fertilizer application rate and irrigation volume in crop water consumption and water use efficiency (WUE).

## 2 Materials and methods

### 2.1 Site decription

**Figure 1:**
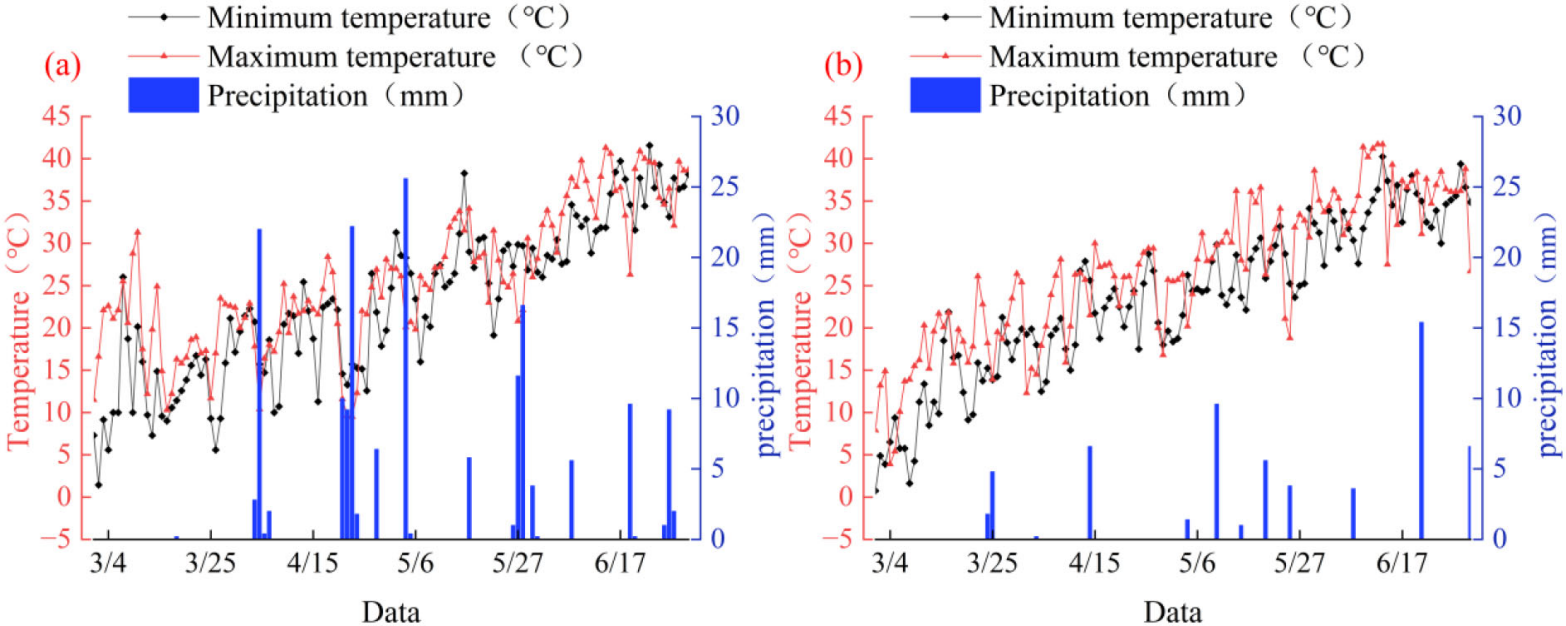
Weather patterns during the growing season of maize/mung bean in 2023 and 2024: weather patterns in 2023 **(a)**, weather patterns in 2024 **(b)**.

The experiment was conducted from March 2023 to July 2024 at the Comprehensive Experimental Station of Hebei Agricultural University, located in Xingtai City, Hebei Province, China (37°34′N, 115°13′E). The region experiences a continental monsoon climate, characterized by an average annual temperature of 14.8°C, annual precipitation of 748.5 mm, and 2235 hours of sunshine. The soil at the site is yellow loam, with a bulk density of 1.41 g cm^-3^, pH 7.5, and the nutrient properties (0 – 30 cm depth): organic matter (12.09 g kg^-1^), total nitrogen (0.73 g kg^-1^), alkali-hydrolyzable nitrogen (131 mg kg^-1^), available phosphorus (20.5 mg kg^-1^), and available potassium (121 mg kg^-1^). Meteorological data for temperature and precipitation during the experimental years (2023 and 2024) are provided in **Figure 1**.

The trial periods spanned 109 days (8 March – 25 June 2023) and 108 days (15 March – 1 July 2024). To ensure optimal growth conditions, maize and mung bean were cultivated under plastic film mulching, which was removed on 20 April 2023 and 30 April 2024, respectively. Pest management included foliar applications of cypermethrin (for maize) and bifenthrin (for mung bean) as required. Pre-emergence weed control was achieved through metolachlor application prior to seeding, supplemented by manual weeding during the growing season.

### 2.2 Experimental design

The farming habits of local farmers are applying compound fertilizer and urea at 1500 ~ 2000 kg·ha^-1^ for maize and 600 ~ 900 kg·ha^-1^ for mung beans The irrigation method was border irrigation, with each irrigation to field capacity (FC). Therefore, according to the local practice, the application of chemical fertilizer was reduced, organic fertilizer was used as part of the nitrogen source input, and border irrigation was changed to different irrigation levels controlled by the upper and lower limits of FC. Split plot design was adopt double factors, crack area for water level: 40%θ_FC_<θ<60%θ_FC_ (I0), 60%θ_FC_<θ<80%θ_FC_ (I1), 80%θ_FC_<θ<95%θ_FC_ (I2); The main area is the application amount of organic fertilizer: Organic fertilizer (F0, 0 kg·ha^-1^) was not applied, the recommended application amount was reduced by 50% (F1, 3750 kg·ha^-1^) and the recommended application amount (F2, 7500 kg·ha^-1^). Organic fertilizer was consisted of locally sourced decomposed chicken manure (organic matter content 14.5 g·kg^-1^, total nitrogen content 20 g·kg^-1^). Total phosphorus content 18 g·kg^-1^, total potassium content 9 g·kg^-1^, water content 320 g·kg^-1^). There were 9 treatments with 3 repetitions for each treatment, and a total of 27 spots with an area of 3.5 m × 6 m.

Maize and mung bean intercropping 4M6B (4 rows of maize: 6 rows of mung bean) as planting method, maize strip width 1.5 m, mung bean strip width 2.0 m, maize and mung bean belt spacing 0.6 m. The mung bean variety was Jilu 20, which came from the Grain and Oil Crops Research Institute of Hebei Academy of Agriculture and Forestry Sciences. The row spacing of mung bean plants was 0.1 m × 0.4 m, and the seeding density was 162,000 plants·ha^-1^. The maize variety was Jinguan 220 (fresh maize) from Beijing Sihai Seed Industry Co., LTD., with row spacing of 0.2 m × 0.5 m and seeding density of 36,000 plants·ha^-1^. Detailed water and nutrient inputs are shown in **Table 1**. Maize and mung bean were harvested on June 28 in both years.

**Table 1:** Water and nutrient inputs of maize/mung bean under different treatments.

Treatment	Organic fertilizer (kg/hm^2^)	Inorganic fertilizer (kg/hm^2^)						Nutrient input (kg/hm^2^)						Field moisture capacity		Effective precipitation (mm)		Irrigating quota on each application (mm)				Irrigation water quantity (mm)			
		Maize			Mung bean			Maize			Mung bean							Maize		Mung bean		Maize		Mung bean	
		N	P_2_O_5_	K_2_O	N	P_2_O_5_	K_2_O	N	P_2_O_5_	K_2_O	N	P_2_O_5_	K_2_O	Upper limit	floor	2023	2024	2023	2024	2023	2024	2023	2024	2023	2024
F_0_I_0_	0	198	60	60	0	0	0	198	60	60	0	0	0	θ_60%_	θ_40%_	166.40	57.00	0	75.00	0	46.87	166.40	132.00	166.40	103.87
F_0_I_1_	0	198	60	60	0	0	0	198	60	60	0	0	0	θ_80%_	θ_60%_	166.40	57.00	194.24	178.12	113.56	78.12	360.64	235.12	279.96	135.12
F_0_I_2_	0	198	60	60	0	0	0	198	60	60	0	0	0	θ_95%_	θ_80%_	166.40	57.00	272.24	209.38	152.52	93.75	438.64	266.38	318.92	150.75
F_1_I_0_	3750	198	60	60	0	0	0	249	106	83	75	68	34	θ_60%_	θ_40%_	166.40	57.00	0	84.38	0	62.50	166.40	119.50	166.40	119.50
F_1_I_1_	3750	198	60	60	0	0	0	249	106	83	75	68	34	θ_80%_	θ_60%_	166.40	57.00	211.26	240.63	96.64	109.38	377.66	297.63	263.04	166.38
F_1_I_2_	3750	198	60	60	0	0	0	249	106	83	75	68	34	θ_95%_	θ_80%_	166.40	57.00	321.55	271.89	90.76	125.01	487.95	325.89	257.16	182.01
F_2_I_0_	7500	198	60	60	0	0	0	300	152	106	150	135	68	θ_60%_	θ_40%_	166.40	57.00	0	89.38	0	71.88	166.40	146.38	166.40	128.88
F_2_I_1_	7500	198	60	60	0	0	0	300	152	106	150	135	68	θ_80%_	θ_60%_	166.40	57.00	238.76	303.13	116.35	140.63	405.16	360.13	282.75	197.63
F_2_I_2_	7500	198	60	60	0	0	0	300	152	106	150	135	68	θ_95%_	θ_80%_	166.40	57.00	340.27	334.38	157.08	156.25	506.67	391.38	323.48	213.25

Since the nitrogen content of organic fertilizer could not fully meet the growth demand of maize, quantitative chemical fertilizer was applied to maize sowing, including 375 kg·ha^1^(N-P_2_O_5_-K_2_O: 16-16-16) compound fertilizer and 300 kg·ha^1^ urea (N≥46.0%), and only organic fertilizer was applied to mung bean sowing. After fertilizing maize and mung bean, rotary tillage was carried out, and the depth of rotary tillage was 0.3 m. Under the I0 irrigation level, there was no excess irrigation except effective precipitation in 2023 during the whole growth period of maize and mung bean, and one irrigation was carried out in 2024. At the I1 irrigation level, maize and mung bean were irrigated once in 2023 and twice in 2024. At the I2 irrigation level, maize and mung beans were irrigated twice in 2023 and three times in 2024. According to the formula of irrigation quota, the planned depth of wet layer for maize and mung bean during irrigation is 0.6 m (drawing stage) and 0.8 m (filling stage) respectively, and the planned depth of wet layer for mung bean is 0.2 m (branching stage) and 0.4 m (flowering pod stage) respectively. The irrigation method is pipeline irrigation, and the irrigation quota is controlled by solenoid valves.

### 2.3 Measurements

#### 2.3.1 Leaf area index

In 2023 and 2024, three maize and mung bean plants were randomly selected from each plot at 35, 55, 70, 85, and 102 d after maize emergence and at 40, 55, and 70 d after mung bean emergence, and the leaf area index (LAI) was calculated by determining the functional leaf length (*L*_ij_) versus the maximum width (*B*_ij_) of each plant using a straightedge.


(1) $$\text{LAI}=0.75\rho \frac{\sum_{j=1}^{m}\sum_{i=1}^{n}{\text{(L}}_{ij}\times {\text{B}}_{ij})}{\text{m}}$$

where *n* is the total number of leaves of plant *j*; m is the number of plants measured; and *ρ* is the planting density.

#### 2.3.2 Chlorophyll relative content

A hand-held dual-wavelength chlorophyll meter (SPAD-502, Minolta Camera Co, Ltd,Japan) was used in 2023 and 2024 at 35, 55, 70, 85, and 102 d after seedling emergence of maize and at 40, 55, and 70 d after seedling emergence of mung bean. Three maize and mung bean plants in side rows were selected in each plot, and Chlorophyll relative content (SPAD) was measured and finally averaged for all leaves of the whole plant, excluding damaged or wilted leaves and avoiding large leaf veins. Measurements were taken at the same time as the photosynthetic parameters, between 9:00 and 11:00 a.m. in the field.

#### 2.3.3 Indicators of photosynthetic parameters

Yaxin-1102g portable photosynthetic apparatus (Beijing Yaxin Riyi Technology Co., Ltd., Beijing, China) was used for 35, 55, 70, 85 and 102 d after maize emergence and 40, 55 and 70 d after mung bean emergence, 9:00 am to 11:00 am: Three maize and mung bean plants were selected from each plot to detect Pn (μmol·m^-2^·s^-1^), transpiration rate (Tr, mmol·m^-2^·s^-1^), stomatal conductance (Gs, mmol·m^-2^·s^-1^) and intercellular CO_2_ concentration (Ci, μmol·mol^-1^). The photosynthetic performance of maize was measured by the first fully unfolded leaf from top to bottom from 35 d to 70 d, and by ear leaf from 70 d to 102 d. The first compound leaf was taken from Mung bean.

#### 2.3.4 Absorbance

Yaxin-1201 Plant Canopy Meter (Beijing Yaxin Riyi Technology Co., Ltd., Beijing, China) is an effective tool for quantitatively describing the structural parameters of plant canopies. The image method was chosen to obtain the canopy structure image. And by borrowing the principle of Beer’s law, the universally recognized semi-empirical and semi-theoretical mathematical modeling formulas were used to non-destructively profile the parameters such as LAI, scattered radiation coefficients, and canopy porosity of the canopy leaves. absorbance (Ab) of maize was photographed using Yaxin-1201 plant canopy meter at 35, 55, 70, 85 and 102 d after emergence of maize for monitoring in synchronization with photosynthetic parameters.

#### 2.3.5 Soil water storage and evapotranspiration

Before seeding and at the end of each growth period, 3 points were randomly selected in each plot, and the sampling position was between the peer plants. Use a soil drill to take a soil sample every 10 cm in the 0 ~ 100 cm soil layer. The soil sample taken from the field is immediately stored in a closed aluminum box. The drying method was used to bake the soil in the oven at 105 °C for 48 hours to constant weight and then weighed it. The formula for calculating soil moisture content SWS and ET was as follows:


(2) $${\text{b}}_{i}=\frac{{\text{w}}_{i}-{\text{d}}_{i}}{{\text{d}}_{i}-{\text{v}}_{i}}$$

In the formula, *b_i_* is the soil moisture content of layer i (%),*w_i_* is the wet soil weight of layer i (g), *ρ_i_* is the dry soil weight of layer i (g), *v_i_* is the aluminum box weight of layer i (g).


(3) $$\text{SWS=}\sum_{1}^{n}{\text{h}}_{i}\times \rho_{i}\times {\text{b}}_{i}$$

where *SWS* is the soil water storage (mm), *h_i_* is the depth of the i soil layer (cm), *ρ_i_* is the soil bulk density of the i soil layer (g·cm^-3^), *b_i_* is the mass water content of the i soil layer (%), and *n* is the number of soil layers.


(4) $$\text{ET}=\text{P}+{\text{I}}_{i}+{\text{(SWS}}_{i}-{\text{SWS}}_{i+1})$$

In the formula, *ET* is evapotranspiration (mm), *P* is precipitation during growth period (mm), *I_i_* is treated irrigation amount (mm), *SWS_i_* is soil water storage in 0 - 100 cm soil layer at the end of the previous growth period (mm), *SWS_i_*_+1_ is soil water storage in 0 - 100 cm soil layer at the end of growth period (mm).

#### 2.3.6 Yield water use efficiency

WUE was calculated as


(5) $$\text{WUE}=\frac{Y}{ET}$$

where WUE is water us efficiency *Y* is plant yield (kg·ha^-1^) and *ET* is evapotranspiration (mm).

#### 2.3.7 Dry matter accumulation and yield

For dry matter accumulation (DMA) measurements of maize and mung bean, three plants each were randomly selected from each plot after maize and mung bean harvest and brought back to the chamber. The maize and mung bean were placed in paper bags and dried in an oven at 80°C for 48 h to a constant weight. The dry weight of the plants was subsequently determined. Maize and mung bean yields were hand harvested for maize and mung bean seed yields on June 25, 2023 and July 1, 2024, respectively. When harvesting maize blocks, the actual effective number of plants and the actual number of ears per maize plant in each treated maize belt were investigated, and the maize plants without long ears were not counted as effective trees. Ten ears were randomly selected from each plot to investigate the fresh weight of maize ears, and the fresh food yield was calculated by multiplying the weight of fresh ears without bracts and the number of effective plants. For mung bean, 1 m × 1 m blocks were selected for each plot, and the number of plants, pods per plant, graminous per pod, and 100 - kernel weight of mung bean were examined and theoretical yields were calculated.

### 2.4 Statistical analysis

Microsoft Excel 2019 and SPSS 25.0 were used for statistical analysis. The effects of LAI, SPAD, photosynthetic characteristics, DMA, ET, WUE and yield on emergence time, organic fertilizer application amount, irrigation amount and planting years of maize and mung bean were investigated by multivariate analysis of variance. The least significant difference (LSD) was used for ANOVA and multiple comparison (*P*<0.05). The correlation between Pn, SPAD and Ab was firstly transformed into dimensionless numbers ranging from 0 to 1 by normalization, then curves were estimated by SPSS, nonlinear fitting was applied to solve equation parameters, and nonlinear curve fitting was performed by Origin 2024 regression analysis. All other graphics were mapped using Origin 2024 software.

## 3 Results

### 3.1 Effects of different water and fertilizer treatments on dry matter accumulation of corn and mung beans

**Figure 2:**
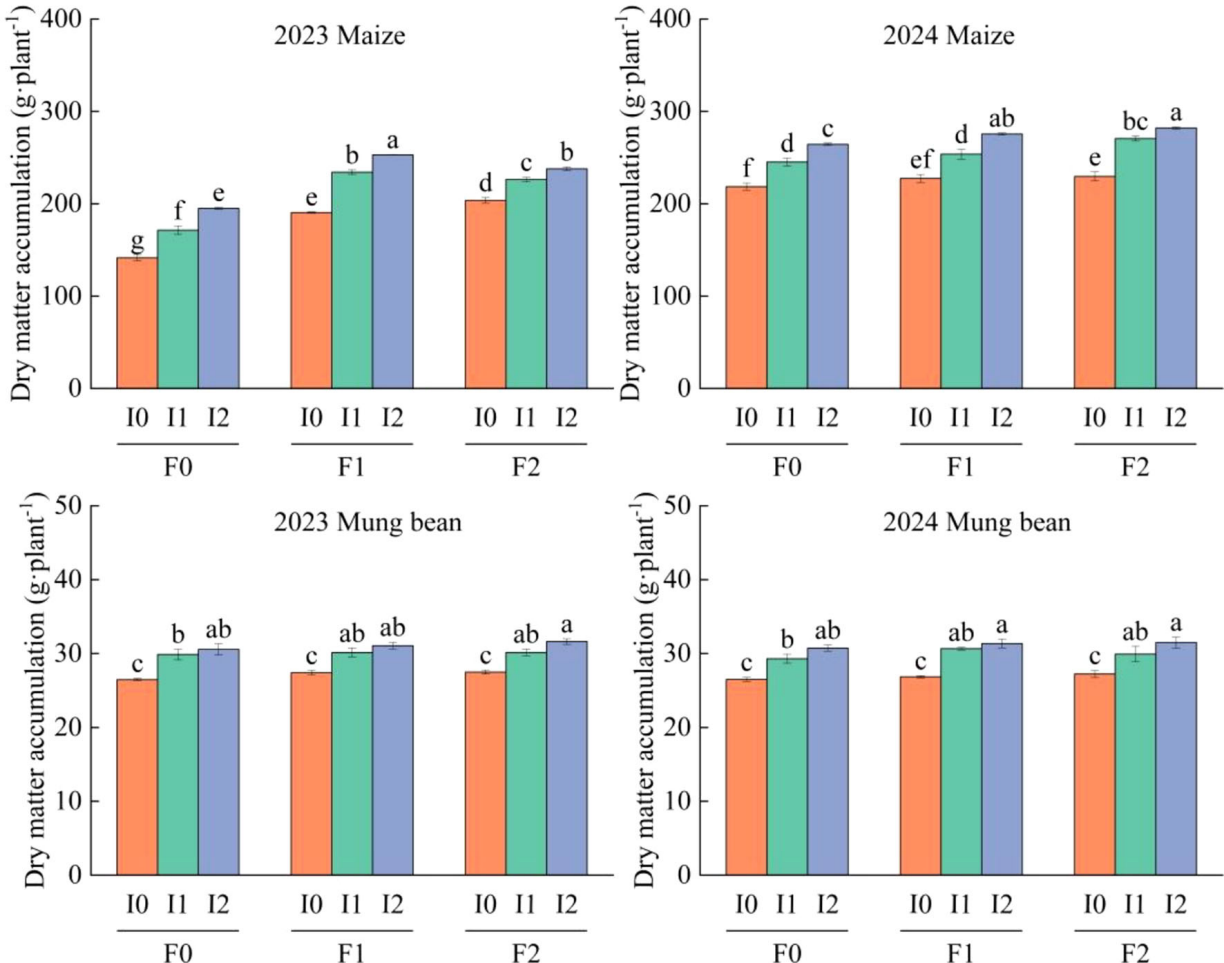
DMA of maize/mung bean under different organic fertilizer application and irrigation treatments in 2023 and 2024. F0 ~ F2 represents different organic fertilizer application rates, and I0 ~ I2 represents different irrigation rates. The two-factor analysis of variance method and post-test were performed using the LSD method; the lowercase letters on the column represent significant differences (*P*<0.05) under different organic fertilizer and irrigation water treatments.

Irrigation volume, organic fertilizer application rate, and their interactions significantly affected the DMAof both maize and mung bean (*P*<0.05), while planting year also exerted significant effects on DMA (*P*<0.05; **Table 2**). Over the two-year study, irrigation had a larger effect on DMA than organic fertilizer application for both crops (**Figure 2**). For mung bean, DMA under I1 and I2 irrigation levels (strip irrigation) was significantly higher than I0 (*P*<0.05), but no significant differences were observed between I1 and I2 (*P*>0.05). Similarly, DMA in F1 and F2 organic fertilizer treatments exceeded F0 (*P*<0.05), with no notable differences between F1 and F2 (*P*>0.05). In maize strips, DMA exhibited a progressive increase with elevated irrigation and organic fertilizer inputs (*P*<0.05), contrasting with mung bean strips where such trends were absent.

**Table 2:** Results of three-factor analysis of variance for photosynthetic physiological characteristics of maize/mung bean [planting age (Y), amount of organic fertilizer (F) and irrigation (I)].

Treatment	Pn		Tr		Gs		Ci		SPAD		LAI		Ab		DMA		Yield	
	Maize	Mung bean	Maize	Mung bean	Maize	Mung bean	Maize	Mung bean	Maize	Mung bean	Maize	Mung bean	Maize	Mung bean	Maize	Mung bean	Maize	Mung bean
Y	1.91ns	49.80**	12.08**	64.40**	6.27*	910.05**	0.24ns	18.83**	77.39**	81.21**	2.05ns	1.59ns	4.86*	–	991.30**	61.55**	0.11ns	27.74**
F	25.86**	35.80**	182.01**	114.18**	25.85**	45.30**	30.21**	15.61**	48.43**	43.41**	109.16**	5.75**	45.79**	–	247.48**	115.13**	3.66*	31.35**
I	263.67**	363.07**	1041.73**	852.69**	68.02**	1112.79**	81.01**	347.17**	190.60**	500.20**	789.30**	55.78**	24.53**	–	393.60**	116.02**	98.13**	156.15**
Y×F	0.67ns	3.19ns	3.98*	0.76ns	1.22ns	10.12**	1.51ns	2.84ns	0.50ns	1.23ns	0.74ns	0.17ns	0.13ns	–	93.91**	15.61**	0.12ns	0.14ns
Y×I	0.99ns	9.29**	4.11*	7.77*	3.37*	9.54**	0.13ns	0.36ns	1.02ns	0.94ns	33.36**	0.87ns	0.27ns	–	0.06ns	6.71**	0.19ns	2.02ns
F×I	7.95**	2.93*	19.22**	8.56**	1.76ns	8.46**	5.31*	7.41**	0.76ns	15.24**	1.79ns	0.12ns	2.24ns	–	2.66*	6.61**	0.26ns	4.56**
Y×F×I	0.64ns	1.37ns	0.63ns	3.71*	0.06ns	4.49**	0.06ns	0.92ns	0.86ns	0.20ns	1.16ns	0.12ns	0.20ns	–	5.55**	1.69ns	0.33ns	0.46ns

ns means no significant difference (*P*>0.05); * and ** means significant difference at *P*<0.05 level or at *P*<0.01 level, respectively.

Quantitatively, mung bean DMA ranged from 26.47 to 31.36g·plant^-1^. Relative to F0, F1 and F2 treatments increased DMA by 1.98 ~ 2.73% and 2.47 ~ 2.72%, respectively. Compared to I0, I1 and I2 irrigation levels enhanced DMA by 10.78 ~ 11.53% and 14.60 ~ 16.13%, respectively. Maize DMA varied between 179.99 and 294.30 g·plant^-1^. F1 and F2 treatments elevated DMA by 3.91 ~ 33.34% and 7.45 ~ 31.47%, respectively, relative to F0, while I1 and I2 increased DMA by 13.91 ~ 18.00% and 21.71 ~ 28.07%, respectively, compared to I0 (**Figure 2**).

### 3.2 Effects of different water and fertilizer treatments on yield of corn and mung beans

Both maize and mung bean yields were significantly influenced by irrigation volume and organic fertilizer application rate (*P*<0.05). A significant interaction effect between these factors was observed for mung bean (*P*<0.05), but not for maize (*P*>0.05). Planting year significantly affected mung bean yield (*P*<0.05), whereas no such effect was detected for maize (*P*>0.05; **Table 2**).

Over the two-year study, mung bean yield under the F0I0, F0I1, and F0I2 treatments exhibited significant differences (*P*<0.05), with yields progressively increasing alongside irrigation volume. In F1 and F2 treatments, I1 and I2 irrigation levels significantly surpassed I0 (*P*<0.05), yielding 16.23 ~ 21.27% and 23.55 ~ 27.49% increases, respectively. However, no significant differences were observed between I1 and I2 (*P*>0.05). Similarly, mung bean yields under F1 and F2 treatments significantly exceeded F0 (*P*<0.05), showing 8.78 ~ 9.10% and 9.51 ~ 10.47% enhancements, respectively.

**Figure 3:**
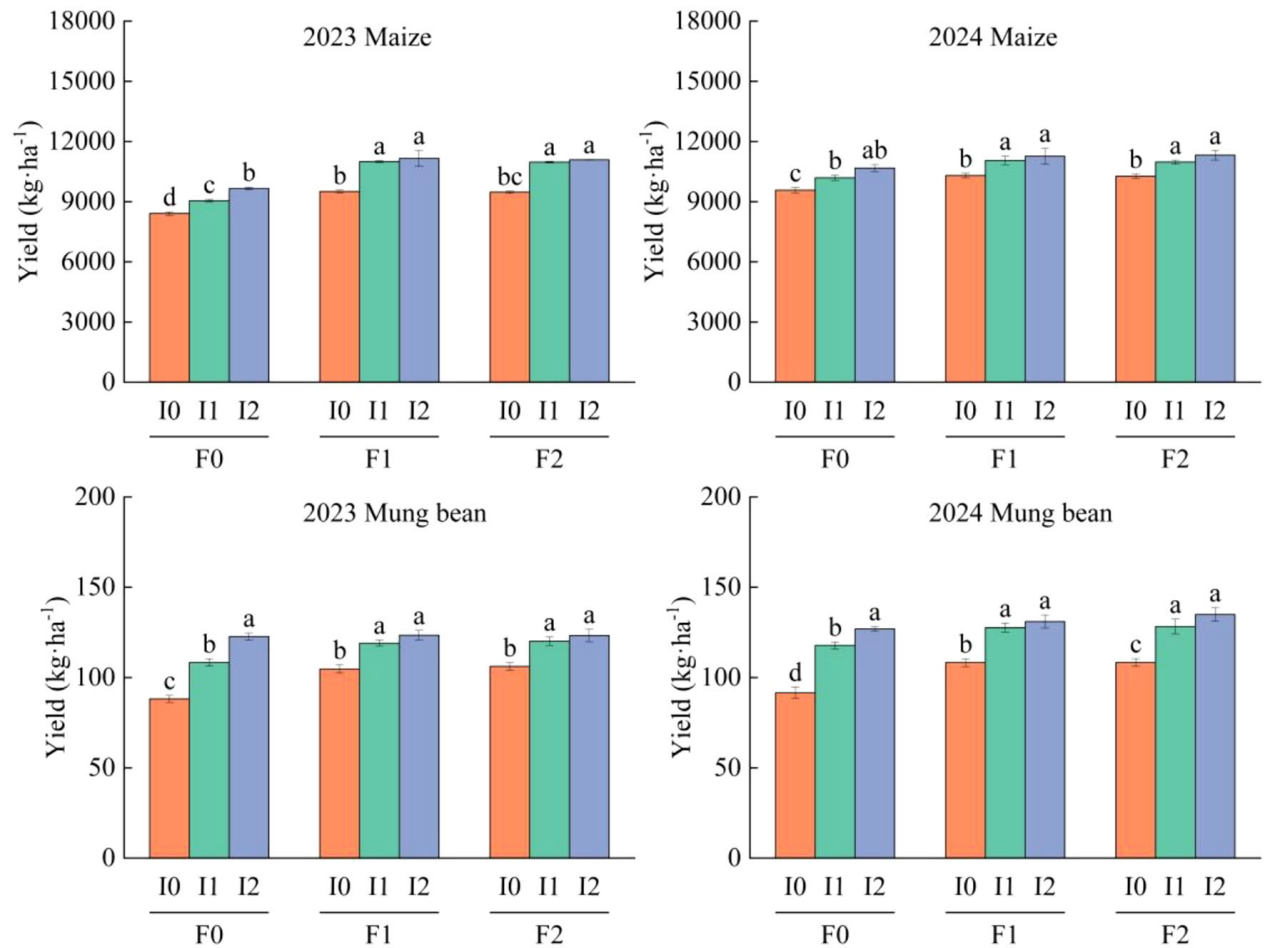
Yield of maize/mung bean under different organic fertilizer application rates and irrigation water treatments in 2023 and 2024. F0 ~ F2 represents different organic fertilizer application rates, and I0 ~ I2 represents different irrigation water rates. The two-factor analysis of variance method and post-test were performed using the LSD method; the lowercase letters on the column represent significant differences (*P*<0.05) under different organic fertilizer amounts and irrigation amounts.

For maize, yields in F1 and F2 treatments were 7.18 ~ 16.73% and 6.98 ~ 16.22% higher than F0, respectively. Irrigation treatments followed analogous trends: I1 and I2 levels increased yields by 6.89 ~ 13.25% and 10.33 ~ 16.48%, respectively, compared to I0 (**Figure 3**).

### 3.3 Effects of different water and fertilizer treatments on leaf area index of corn and mung beans

#### 3.3.1 Maize

Maize LAI peaked between 70 and 85 days (d) after emergence (*P*<0.05), with no interannual differences (*P*>0.05; **Table 3**). Three-way ANOVA (**Table 2**) revealed significant main effects of irrigation volume and organic fertilizer application rate on LAI (*P*<0.05), whereas their interaction and planting year exhibited no significant impacts (*P*>0.05).

**Table 3:** Results of the two-factor analysis of the photosynthetic physiological characteristics of maize with the emergence time and planting age, Lowercase letters indicate results with *P*<0.05 significance at different irrigation levels.

Planting year	Days after sowing	LAI	SPAD	Pn (μmol m^-2^ s^-1^)	Tr (mmol m^-2^ s^-1^)	Gs (mmol m^-2^ s^-1^)	Ci (μmol mol^-1^)	Ab
2023	35 d	1.06d	47.64c	22.22d	5.91c	261.73d	147.23d	0.68d
	55 d	1.90c	61.58a	42.38b	6.97b	313.49b	211.07b	0.89b
	70 d	2.46b	61.22a	45.92a	8.07a	338.56a	287.08a	1.01a
	85 d	3.07a	50.91b	31.56c	5.48c	332.76a	277.63a	0.78c
	102 d	3.05a	41.11d	17.50e	3.06d	200.46c	180.21c	0.62e
2024	35 d	1.11e	45.68d	21.39d	6.08c	263.57c	145.02d	0.68d
	55 d	1.89d	64.02a	42.40b	7.01b	302.39b	240.53b	0.91b
	70 d	2.57c	59.68b	45.36a	8.05a	329.76a	274.20a	1.04a
	85 d	2.93b	49.32c	30.64c	5.58d	304.06b	250.58b	0.79c
	102 d	3.13a	36.18d	18.30e	3.22e	218.06d	187.76c	0.64e
Year		0.22ns	26.75**	0.51ns	0.83ns	4.17*	0.11ns	3.96*
Time		372.88**	1221.61**	715.79**	260.03**	252.11**	269.36**	399.10**
Year×Time		1.33ns	21.33**	0.61ns	0.13ns	7.16**	9.88**	0.54ns

ns means no significant difference (*P*>0.05); * and ** means significant difference at *P*<0.05 level or at *P*<0.01 level, respectively.

Specifically, compared to the control (F0), F1 and F2 organic fertilizer treatments increased LAI by 5.99 ~ 7.80% and 12.68 ~ 36.70%, respectively. F2 further enhanced LAI by 6.96 ~ 24.27% relative to F1. Irrigation treatments followed similar trends: I1 and I2 elevated LAI by 9.73 ~ 11.16% and 14.49 ~ 33.42%, respectively, compared to I0, with I2 surpassing I1 by 8.12 ~ 11.22% (**Equation 1**).

#### 3.3.2 Mung bean

**Figure 4:**
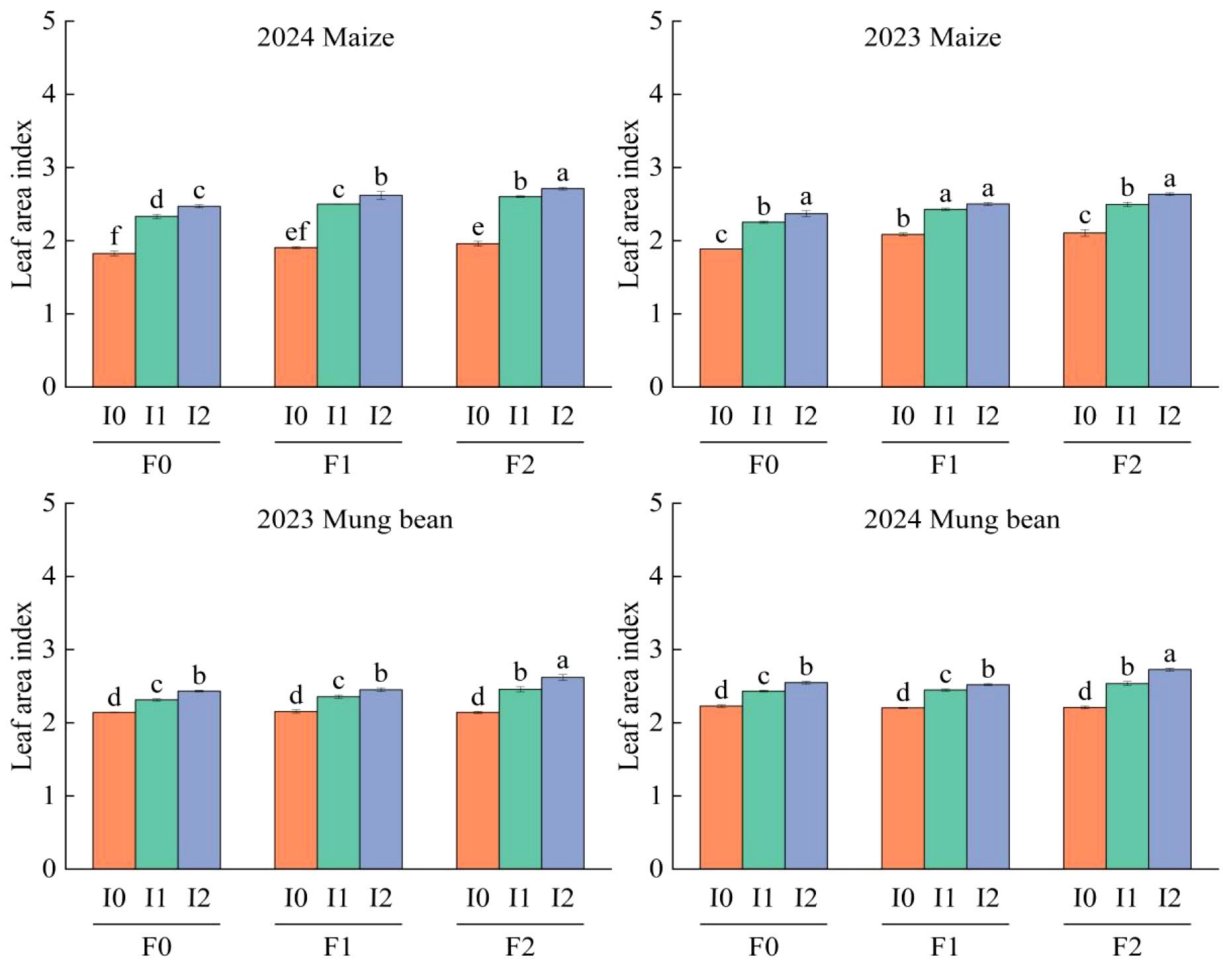
LAI of maize/mung bean under different organic fertilizer application rates and irrigation amounts in 2023 and 2024. F0 ~ F2 represents different organic fertilizer application amounts, I0 ~ I2 represents different irrigation amounts. LSD method was used to conduct two-factor analysis of variance and post-test. Lower case letters on the column represent significant difference under different organic fertilizer amount and irrigation amount (*P*<0.05).

**Table 2** indicates that the quantity of irrigation and organic fertilizer application significantly influenced the LAI of mung bean plants (*P*<0.05). However, the interaction between these two factors did not have a significant effect on the LAI of mung bean (*P*>0.05). Additionally, the influence of planting age on the LAI of mung bean was not significantly different (*P*>0.05). Over a two-year field experiment, the LAI of mung bean exhibited a gradual increase coinciding with the emergence of maize (**Table 4**), reaching its peak within 55 ~ 70 d after emergence (*P*<0.05). Compared to F0, F1 did not show a significant increase (*P*>0.05); F2 increased by 27.43% ~ 28.72%, yet no significant difference was observed between F2 and F0 (*P*>0.05). Similarly, I2 showed an increase of 16.61% ~ 17.39% compared to I0, and there was no significant difference between I1 and I2 (*P*>0.05) (**Figure 4**; **Table 3**).

**Table 4:** Results of the two-factor analysis of the photosynthetic physiological characteristics of mung bean with the emergence time and planting age, Lowercase letters indicate results with *P*<0.05 significance at different irrigation levels.

Planting year	Emergence time	LAI	SPAD	Pn (μmol m^-2^ s^-1^)	Tr (mmol m^-2^ s^-1^)	Gs (mmol m^-2^ s^-1^)	Ci (μmol mol^-1^)
2023	40 d	1.46c	34.25c	21.23a	2.66a	174.06a	325.07a
	55 d	2.56b	44.92a	14.08b	1.87b	161.93b	321.41a
	70 d	3.00a	41.57b	9.84c	1.35c	80.65c	240.97b
2024	40 d	1.57c	34.22c	22.24a	2.84a	193.05a	331.88a
	55 d	2.56b	44.09a	14.48b	2.21b	187.32b	324.48b
	70 d	3.16a	40.86b	10.07c	1.28c	82.26c	242.08c
Year		9.59*	0.87ns	6.41*	1.40ns	40.66**	2.85ns
Time		1056.45**	116.49**	1020.35**	55.60**	737.54**	672.29**
Year×Time		3.08*	0.20ns	1.20ns	1.18ns	8.73**	0.60ns

ns means no significant difference (*P*>0.05); * and ** means significant difference at *P*<0.05 level or at *P*<0.01 level, respectively.

### 3.4 Effects of different water and fertilizer treatments on SPAD of corn and mung beans

#### 3.4.1 Maize

**Figure 5:**
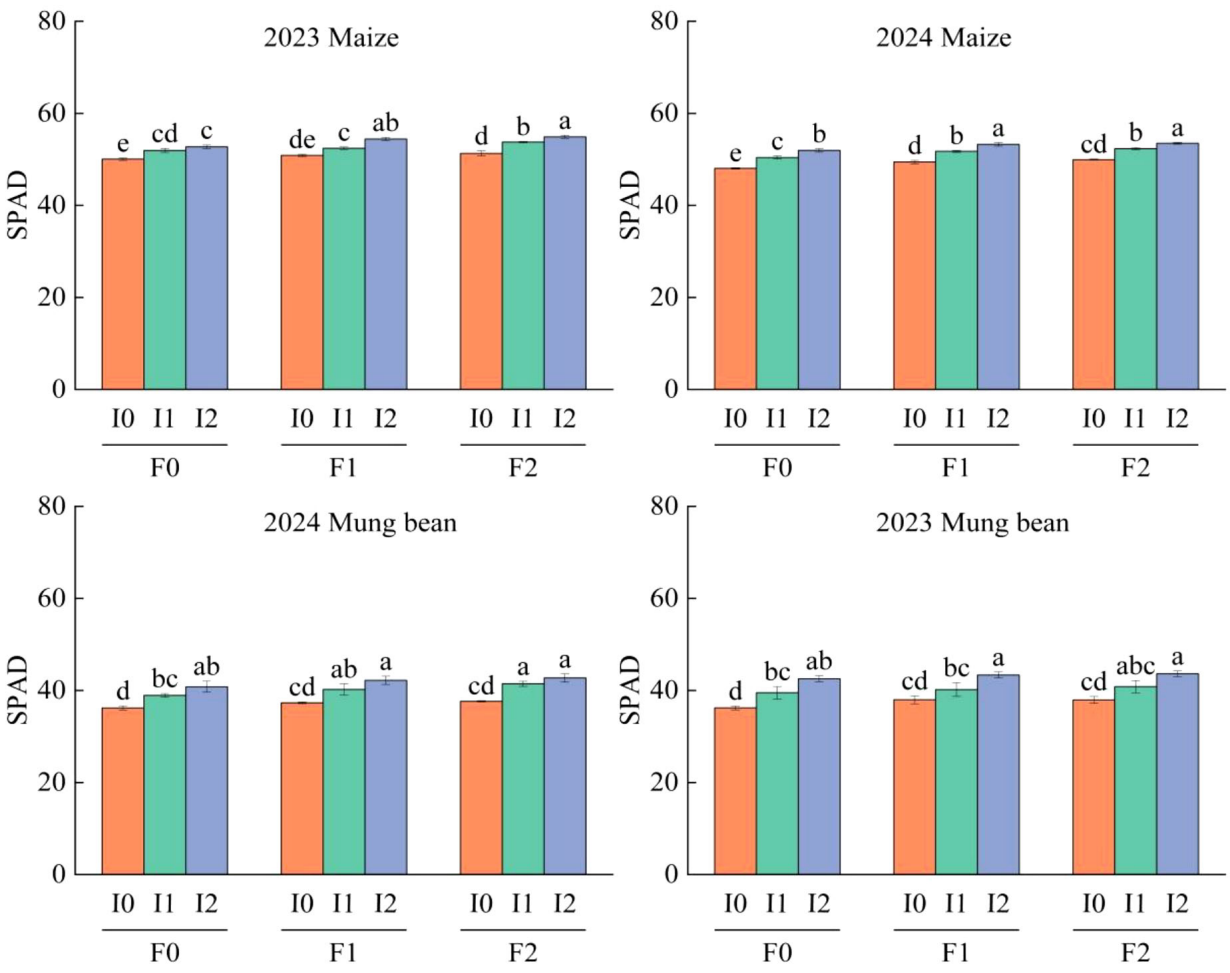
SPAD of maize/mung bean under different organic fertilizer application rates and irrigation amounts in 2023 and 2024. F0 ~ F2 represents different organic fertilizer application amounts, I0 ~ I2 represents different irrigation amounts. LSD method was used to conduct two-factor analysis of variance and post-test. Lower case letters on the column represent significant difference under different organic fertilizer amount and irrigation amount (*P*<0.05).

**Table 2** demonstrates that the quantity of water and organic fertilizer applied had a significant influence on maize SPAD values (*P*<0.05), while their interaction did not exhibit a significant effect (*P*>0.05). Under varying treatments, maize SPAD values progressively increased over time following crop emergence (*P*<0.05) (**Table 5**), ranging from 33.28 ~ 67.40 and reaching a peak between 35 and 70 dafter emergence. F1 exhibited an increase of 1.27% ~ 4.59% compared to F0, whereas F2 showed an increase of 0.55% ~ 4.63% relative to F0, with no significant difference observed between F1 and F2 (*P*>0.05). Maize SPAD values displayed a consistent increasing trend with increasing irrigation levels (*P*<0.05). Specifically, I1 was 0.81% ~ 8.99% higher than I0 from 35 ~ 102 d post-emergence, and I2 was 2.75% ~ 12.68% higher than I0 (**Figure 5**; **Table 3**).

**Table 5:** Water use characteristics of maize/mung bean.

Planting year	Treatment		ET (mm)		Water use effiency (kg ha^-1^ mm^-1^)	
			Maize	Mung bean	Maize	Mung bean
2023	F0	I0	371.09e	260.91c	22.67f	0.34d
		I1	382.66cd	266.14c	23.65ef	0.41bc
		I2	389.83b	271.66b	24.79cde	0.43ab
	F1	I0	378.26c	268.81b	25.14de	0.39c
		I1	386.19bc	271.72a	28.49cde	0.44a
		I2	393.11b	277.54a	28.38bc	0.44a
	F2	I0	380.23cd	275.54b	24.91bcd	0.39c
		I1	392.82b	280.46a	27.93ab	0.43ab
		I2	402.80a	284.60a	27.52a	0.45a
2024	F0	I0	353.77f	267.78e	27.06f	0.34c
		I1	360.85cde	274.64de	28.24ef	0.43a
		I2	363.82cd	281.72abcd	29.34cde	0.45a
	F1	I0	356.11ef	279.61bcd	28.93de	0.39b
		I1	363.87cd	282.51ab	30.39cd	0.45a
		I2	365.44bc	288.32a	30.82bc	0.45a
	F2	I0	359.44de	287.54cde	28.58bc	0.38b
		I1	369.24b	292.70abcd	29.72ab	0.44a
		I2	378.54a	296.71abc	29.90a	0.45a

The letters represent the results of multiple comparisons of the same column of data under *P*<0.05, ns means no significant difference (*P*>0.05); * and ** means significant difference at *P*<0.05 level or at *P*<0.01 level, respectively.

#### 3.4.2 Mung bean

The amount of irrigation, the application rate of organic fertilizer, and their interaction significantly influenced the SPAD values of mung bean (*P*<0.05), as did the planting age (*P*<0.05) (**Table 2**). **Table 5** indicates that under various treatments, the SPAD values of mung bean increased initially and then decreased between 40 and 70 d after emergence (*P*<0.05). Under the main effect of irrigation, the SPAD values for treatments F1 and F2 were significantly higher than those for treatment F0 (*P*<0.05), with no significant difference observed between treatments F1 and F2 (*P*>0.05). Under the main effect of organic fertilizer application, SPAD values gradually increased with increasing irrigation levels. The SPAD values for treatments I2 and I1 were significantly higher than those for treatment I0 (*P*<0.05). Specifically, from 40 to 70 d after emergence, the SPAD values for I1 were 7.09% ~ 8.14% higher than those for I0, while those for I2 were 12.62% ~ 16.85% higher than those for I0, and I2 was 4.14% ~ 9.12% higher than I1 (**Figure 5**; **Table 3**).

### 3.5 Effects of different water and fertilizer treatments on photosynthetic characteristics of corn and mung beans

#### 3.5.1 Maize

**Figure 6:**
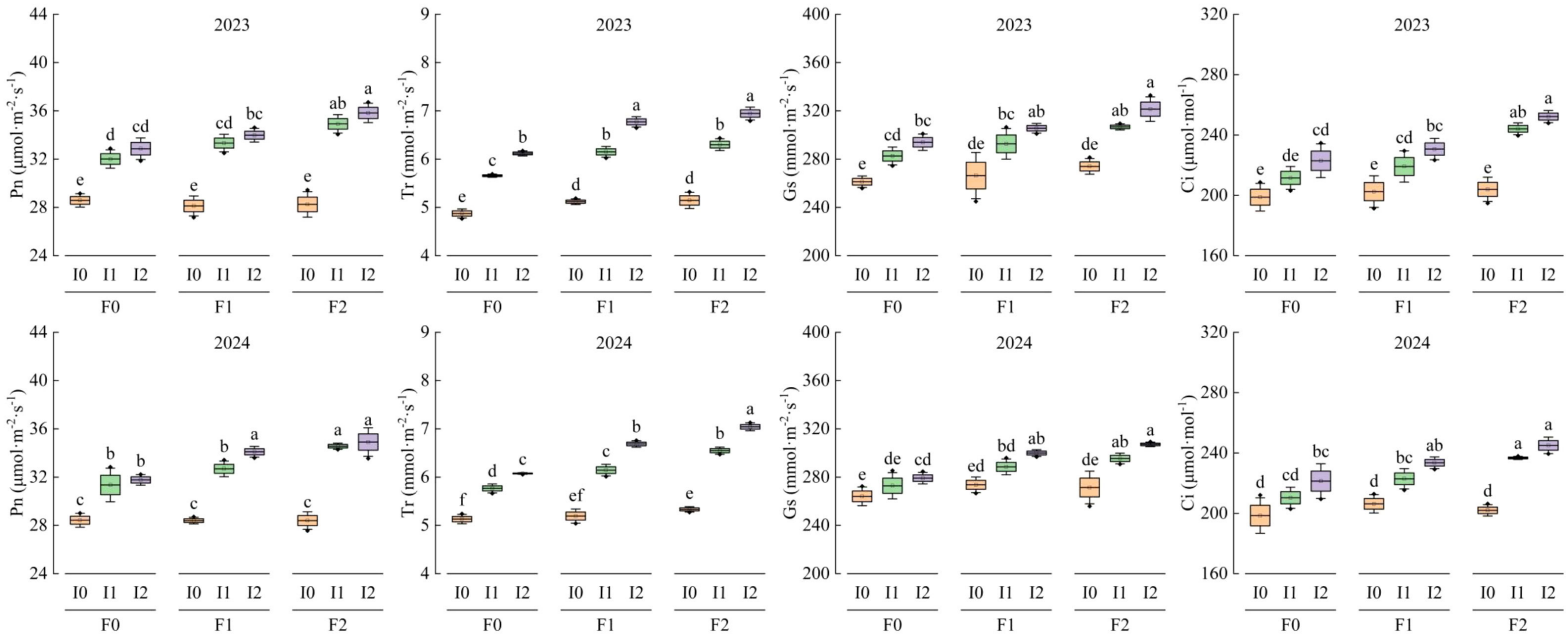
The photosynthetic characteristics of maize under different treatments in 2023 and 2024 with the change of emergence. The box plots show the mean, SD and SE, and the photosynthetic characteristics measured at different emergence times are normally distributed. The black origin represents the data distribution and value, the square box represents the SD, the whiskers represent the mean, and the error bars represent the SE of the total curve sum of the three repeated measurements. F0 ~ F2 represent different organic fertilizer application rates, and I0 ~ I2 represent different irrigation rates. Two-factor analysis of variance was performed using the LSD method and post-tests.

**Table 3** presents the two-factor significant analysis of maize photosynthetic characteristics with respect to emergence time. The emergence time significantly affects Pn, Tr, Gs, and Ci (*P*<0.05), which initially increase and then decrease with emergence time, peaking between 55 and 85 d after emergence. The amount of irrigation water, the application rate of organic fertilizer, and their interaction all significantly influence the photosynthetic characteristics of maize (*P*<0.05), except for Gs (**Table 2**). Under the main effect of irrigation water, the photosynthetic characteristics of treatments F1 and F2 are higher than those of treatment F0. Specifically, the photosynthetic characteristics of treatments I1 and I2 are significantly higher than those of treatment I0. The increases in Pn, Tr, Gs, and Ci for F1 and F2 are 1.09% ~ 9.72%, 0.24% ~ 28.33%, 1.16% ~ 17.33%, and 0.19% ~ 28.84%, respectively. Treatments I1 and I2 enhance photosynthetic characteristics by 5.04% ~ 47.12%, 5.80% ~ 33.33%, 1.97% ~ 25.08%, and 2.69% ~ 33.22%, respectively. Among these, the effect of irrigation on Tr shows a gradual increasing trend (*P*<0.05), whereas no significant differences are observed for Pn, Gs, and Ci. Additionally, there is no significant difference between treatments I1 and I2 (*P*>0.05) (**Figure 6**).

#### 3.5.2 Factors influencing and related to Pn of maize

**Figure 7:**
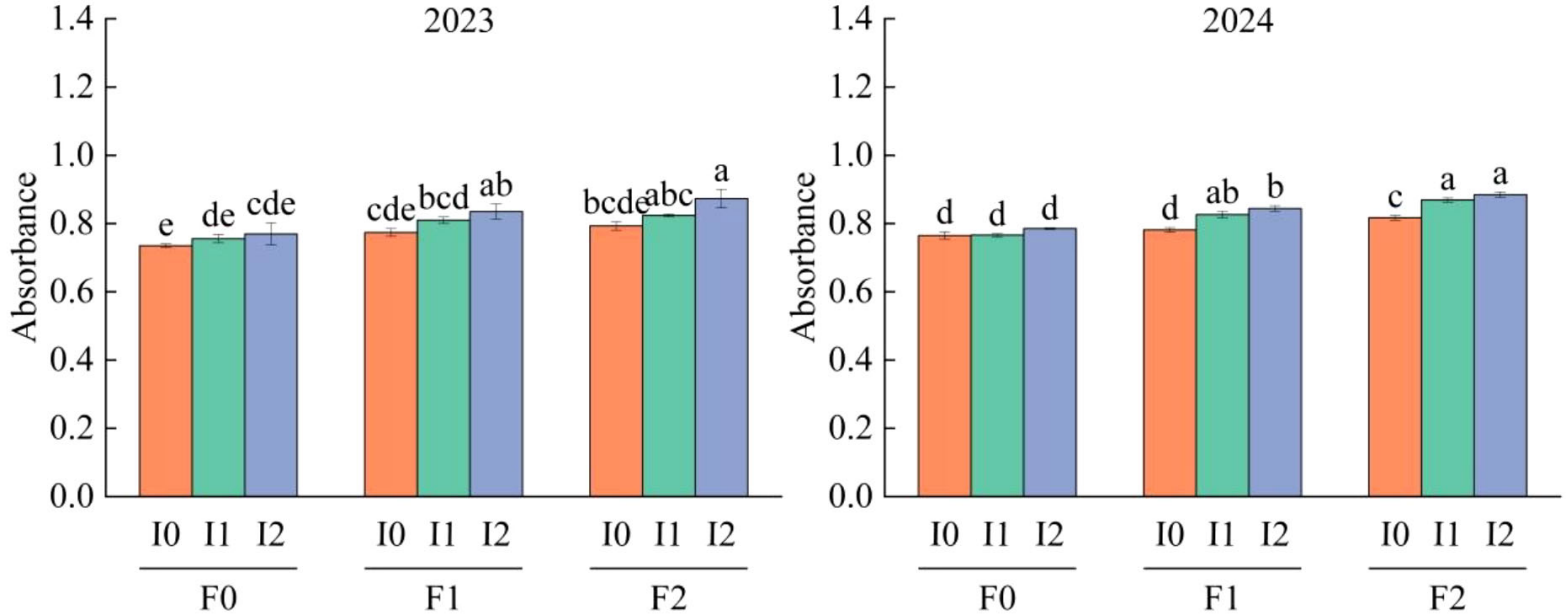
Changes of maize Ab with different organic fertilizer amount and irrigation amount at different seedling times in 2023 and 2024.

Ab could accurately estimate the radiation interception of the dominant crop in both single-crop and mixed-crop systems. Maize seedling emergence time, irrigation amount, organic fertilizer application, and their interactions had significant effects (*P*<0.05) on Ab (**Table 2**). Ab exhibited an increasing and then decreasing trend with advancing maize seedling emergence time, peaking at 55 ~ 80 d after emergence. Additionally, Ab showed a gradual increasing trend with both irrigation amount and organic fertilizer application (**Table 3**). Compared to the F0 treatment, Ab increased by 5.86% ~ 10.13% under F2 and F1 treatments, with an additional increase of 2.95% ~ 3.08% under F2 compared to F1. Similarly, Ab increased by 2.45% ~ 7.66% under I2 and I1 treatments compared to I0, with an additional increase of 3.72% ~ 3.84% under I2 compared to I1 (**Figure 7**).

**Figure 8:**
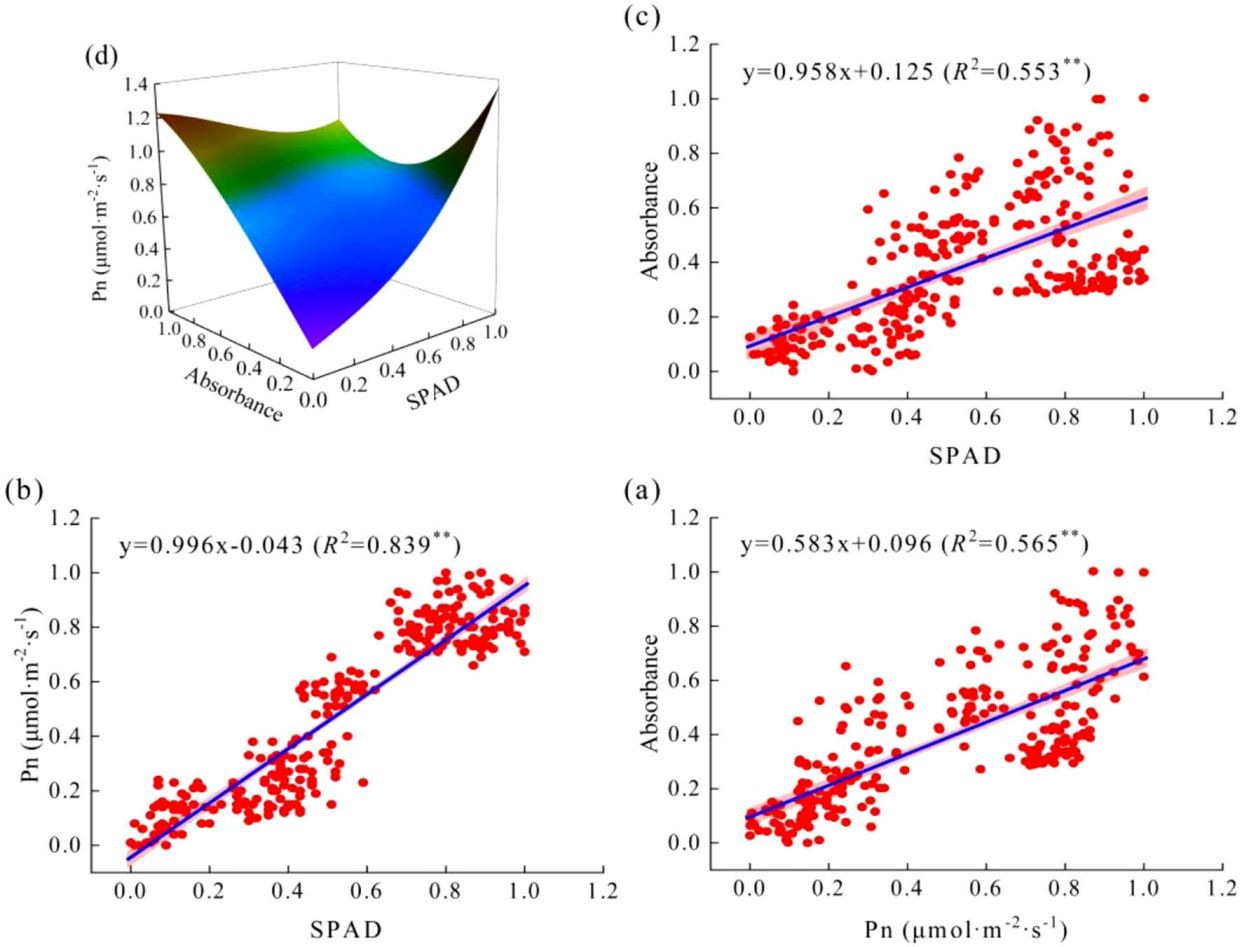
The correlation between Pn, SPAD and Ab of maize and the 3D function images among them. **(a)** is the linear fitting result of Ab and Pn, **(b)** is the linear fitting result of Pn and SPAD, **(c)** is the linear fitting result of Ab and SPAD, and **(d)** is the 3D function fitting image between Pn, SPAD and Ab. The red scatter in the 2D image represents the corresponding data, n=135, the blue line represents the regression equation, and the pink boundary represents the confidence interval, which provides a range of estimates for the population parameters.

Two-year observation data for Pn, SPAD, and Ab were normalized into dimensionless values between 0 and 1. Regression fitting using Origin 2024 software analyzed the relationships among these three variables, yielding the relationship model shown in **Figure 8**. A significant positive correlation was observed between Pn and SPAD, as well as between Pn and Ab (**Figure 8**; *R*²>0.05), reaching a strong correlation level, with all fitted equations achieving significance (*P*<0.05). Among these, the strongest correlation was found between Pn and SPAD (*R*²=0.839), while the correlations between Pn and Ab (*R*²=0.565) and between SPAD and Ab (*R*²=0.553) were relatively weaker. Therefore, SPAD had a stronger influence on Pn compared to Ab. Furthermore, a binary cubic regression model (b) was established for the effects of SPAD and Ab on Pn: $z\;=\;-0.011+0.615x+1.503y-3.016xy-0.754x^{2}y+2.163y^{2}x+0.753x^{3}-0.289y^{3}$ , where x and y represent the respective influence roles of SPAD and Ab on Pn indices.

#### 3.5.3 Mung bean

**Figure 9:**
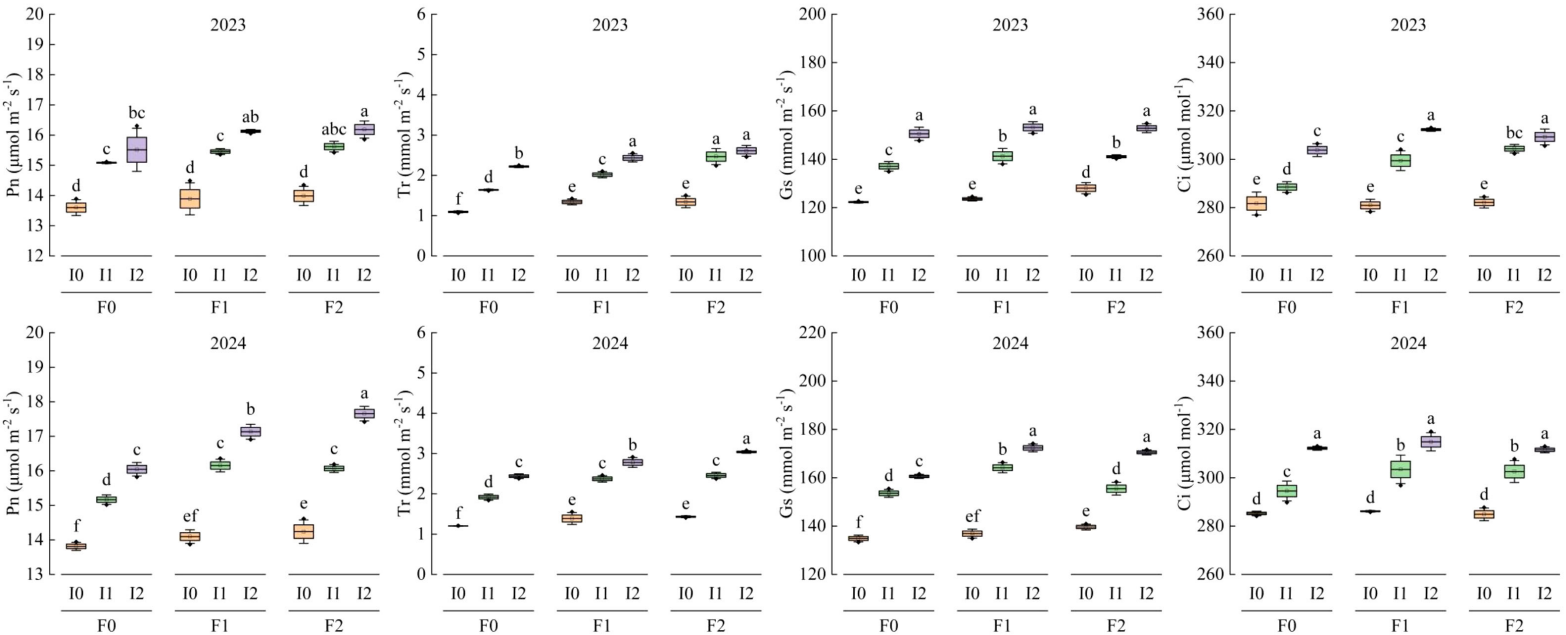
Changes of photosynthetic characteristics of mung bean with seedling emergence under different treatments in 2023 and 2024. The box plot shows the normal distribution of mean value, SD and SE and the photosynthetic characteristics data measured at different seedling emergence times, where the black origin represents the data distribution and value, the square box block represents SD, the required line represents the average value, and the error bar represents the SE of the sum of the total curves measured three times. F0 ~ F2 represents different organic fertilizer application amounts, I0 ~ I2 represents different irrigation amounts. LSD method was used to conduct two-factor analysis of variance and post-test.

The two-factor significant analysis of the photosynthetic characteristics of mung bean with respect to emergence time is presented in **Table 4**. The emergence time of mung bean significantly affects Pn, Tr, Gs, and Ci (*P*<0.05), which initially increase and then decrease with emergence time, peaking between 40 and 70 d after emergence. The amount of irrigation water, the application rate of organic fertilizer, and their interaction had significant effects on the photosynthetic characteristics of mung bean (*P*<0.05), except for Pn (**Tables 2**, **3**). Compared with the F0 treatment, F1 and F2 treatments increased Pn, Tr, Gs, and Ci of mung bean by 0.70% ~ 3.60%, 16.36% ~ 28.93%, 1.98% ~ 5.44%, and 0.79% ~ 2.49%, respectively. However, no significant differences were observed between F1 and F2 treatments (*P*>0.05). The I1 and I2 treatments increased the photosynthetic characteristics of mung bean by 11.29% ~ 20.60%, 0.62% ~ 67.74%, 13.74% ~ 22.37%, and 5.17% ~ 9.61%, respectively, compared with the I0 treatment. The effect of irrigation on the photosynthetic characteristics of mung bean showed a gradual increasing trend (*P*<0.05), which differed from that observed in maize (**Figure 9**).

### 3.6 Effects of different water and fertilizer treatments on ET and WUE of corn and mung beans

#### 3.6.1 Maize

According to the results of the three-factor ANOVA in **Table 2**, planting years, organic fertilizer application amount, and irrigation amount had significant effects on (**Equation 4**) and (**Equation 6**) of maize (*P*<0.05). The interaction between organic fertilizer application amount and irrigation amount significantly influenced ET (*P*<0.05), but did not significantly affect WUE (*P*>0.05). As shown in **Table 5**, with increasing levels of organic fertilizer application and irrigation, ET exhibited a gradually increasing trend (*P*>0.05). However, the pattern of WUE differed; no significant differences in WUE were observed between I1, I2, F1, and F2 treatments (*P*>0.05). Compared with the F0 treatment, ET increased by 0.93% ~ 27.45% under F1 and F2 treatments. Relative to the low irrigation level, ET increased by 2.57% ~ 4.29% under medium and high irrigation levels. Despite the increase in ET, WUE improved significantly: compared with the F0 treatment, WUE increased by 8.61% ~ 10.91% under F1 and F2 treatments. Similarly, relative to the low irrigation level, WUE increased by 7.29% ~ 8.73% under medium and high irrigation levels.

#### 3.6.2 Mung bean

According to the results of three-factor ANOVA in **Table 2**, planting years, organic fertilizer application amount and irrigation amount had significant effects on ET and WUE of mung bean (*P*>0.05). The interaction of organic fertilizer application amount and irrigation amount had significant effects on WUE (*P*<0.05), but had significant effects on ET (*P*>0.05). This is the opposite of what happens with maize. According to **Table 5**, with the increase of organic fertilizer application amount and irrigation level, the effects on ET and WUE of mung bean were the same as those of maize. Compared with F0 treatment, ET in F1 and F2 treatment increased by 2.81% ~ 5.83%. Compared with low irrigation level, ET at medium and high irrigation level increased by 1.70% ~ 3.68%. Compared with F0 treatment, WUE of F1 and F2 treatment increased by 4.13% ~ 5.79%, while WUE of medium and high irrigation level increased by 16.60% ~ 19.74% compared with low irrigation level.

## 4 Discussion

### 4.1 Effect of organic fertilizer application rate and irrigation amount on maize/mung bean intercropping DMA and yield

Many studies have demonstrated that gramineous/legume intercropping exhibits greater potential than monocropping due to interspecies effects (Gao et al., 2024). The aim of this study is to investigate the impact of organic manure, a natural fertilizer, in combination with varying irrigation levels on crop growth and physiology, as well as to analyze the underlying patterns and causes. Consequently, this study did not include a monoculture treatment but directly implemented fertilizer and irrigation treatments. The rules and reasons were analyzed by comparing the effects of no application of organic fertilizer and low irrigation levels as a control.

Rational water-fertilizer combinations can effectively promote crop growth and enhance yield. Both irrigation volume, organic fertilizer application rate, and their interactions significantly influenced the DMA of maize and mung beans (*P*<0.05). Under the same irrigation regime, organic fertilizer application increased DMA and yield in both crops, with no significant differences observed between F1 and F2 treatments (*P*>0.05). Similarly, under the same organic fertilizer level, no significant differences were detected between I1 and I2 treatments (*P*>0.05). These findings align with previous studies (Li et al., 2022; Wu et al., 2023), which reported that optimized water-fertilizer management enhances plant DMA and yield. However, excessive fertilizer application does not lead to further increases in DMA or yield. Additionally, Farooq et al. (2019) demonstrated that substituting chemical nitrogen fertilizers with organic fertilizers improves crop DMA and final yield. Comparing our results with existing literature, it is evident that appropriate organic fertilizer application combined with rational irrigation practices can optimize the growth of intercropped maize and mung beans, thereby increasing productivity.

### 4.2 Effect of organic fertilizer application rate and irrigation amount on LAI and SPAD of intercropped maize/mung bean

This study showed that irrigation amount and application of organic fertilizer had significant effects on LAI and SPAD of maize and mung bean (*P*<0.05), but their interaction had no significant effects on LAI and SPAD of maize and mung bean (*P*>0.05), but had significant effects on SPAD of mung bean (*P*<0.05) (**Table 1**). The LAI and SPAD of maize and mung bean were increased by increasing irrigation amount and applying organic fertilizer. This result is related to soil water content. When soil water is at a low level, plant leaf growth will be inhibited, chlorophyll degradation rate will be accelerated, resulting in leaf degreening and reduced photosynthetic rate; when soil water is at a high level, leaf growth and chlorophyll accumulation will be promoted (Yang et al., 2023b). LAI and SPAD of maize and mung bean showed a trend of increasing first and then decreasing gradually with the emergence time (**Tables 3**, **4**), which was similar to previous results. After maize silk spinning and mung bean flowering, nitrogen in leaves would be transferred to seeds to meet the filling demand of seeds. In addition, with the advance of crop growth period, leaves will gradually age, resulting in a gradual decrease in leaf area index and SPAD, and a phenomenon of leaf greening (Osaki, 1995; Wang et al., 2023; Dosio et al., 2024).

### 4.3 Effect of organic fertilizer application rate and irrigation amount on the photosynthetic performance of intercropped maize/mung bean

The gas exchange parameters of maize gradually increased with higher irrigation levels and organic fertilizer inputs (*P*<0.05) (**Figures 6**, **9**). Across growth stages, photosynthetic traits in maize exhibited a unimodal trend, peaking between 55 – 85 d after emergence (**Table 3**, **Figure 7**). Mung bean showed slightly different patterns: organic fertilization and increased irrigation significantly improved its photosynthetic characteristics (*P*<0.05), but no significant differences were observed between F1F2 or I1I2 treatments (*P*>0.05) (**Figure 9**), indicating that fertilized plants generally outperformed unfertilized ones in photosynthetic rate, though the incremental benefits of higher fertilizer/irrigation levels were marginal. Under low soil moisture, enhanced plant water loss induced stomatal closure to conserve water, consequently reducing Tr, Gs, and Ci (Efthimiadou et al., 2010).

In this study, the high water-fertilizer treatment (F2I2) maintained maize and mung bean photosynthesis in the stomatal limitation domain, characterized by unsaturated Gs, Tr, and Ci across treatments, with continuous Pn improvement. This eliminated non-stomatal limitations (e.g., decoupling of Ci and Pn observed in previous studies; **Figure 6** and **Figure 9**), likely because optimal water and nutrient supply ensured sufficient substrates and energy for photosynthesis. For example (Lo et al., 2019), showed that inadequate water supply reduces electron transport in the initial reaction, limiting CO_2_ uptake and decreasing Pn. In such cases, moderate nitrogen fertilization can significantly enhance leaf SPAD values and Gs, promoting photosynthate accumulation and translocation (Yu et al., 2022). Low nitrogen availability may suppress root nutrient uptake, restricting plant growth and photosynthetic efficiency.

Organic fertilizer enhances leaf function through nutrient supply, while irrigation promotes stomatal opening and nutrient mobilization via stomatal regulation and water-fertilizer coupling, forming a positive feedback loop. Their synergistic effect stabilized photosynthetic performance across years in both crops, ensuring reproducible responses under varying environmental conditions. This study highlights the critical role of balanced water-nutrient management in sustaining photosynthetic efficiency and underscores the importance of distinguishing stomatal vs. non-stomatal limitations in crop physiology research.

### 4.4 The net photosynthetic rate of maize was controlled by absorbance and relative chlorophyll content

LAI, SPAD and available photosynthetic radiation are key factors affecting plant Pn (Tsubo and Walker, 2002; Munz et al., 2014; Bonelli and Andrade, 2020). Ab is a physical quantity used to measure the degree of light absorption, which can directly reflect the ability of the blade to absorb light (Chen et al., 2023a). In this study, only maize Ab was measured and analyzed, because mung bean plants were short and the distance from the surface was limited, and the measurement of Ab was inaccurate. maize Ab showed a single-peak trend with the advancement of maize growth period, and gradually increased with the increase of irrigation amount and organic fertilizer amount, which was similar to the change law of physiological characteristics of maize leaves in the previous paper. In the previous paper, we concluded that appropriate soil water management and increasing the application amount of organic fertilizer could promote LAI and SPAD, thus promoting the light utilization ability of leaves. Moreover, according to the correlation analysis results of Pn, Ab and SPAD, it can be seen that Pn, SPAD and Ab are positively correlated with each other, and all of them have reached a significant level (P<0.05). According to the three-dimensional function model established by Pn, Ab and SPAD, Namely $z=-0.011+0.615x+1.503y-3.016xy-0.754x^{2}y+2.163y^{2}x+0.753x^{3}-0.289y^{3}$ , It shows that Pn increases with the action of SPAD and Ab in S-shaped curve, which increases first and then decreases. This further confirms the above mentioned that LAI and SPAD of maize leaves decrease after silking with the advancement of the growth period, and the aging of maize leaves leads to the decrease of light absorption capacity of maize. With the increase of organic fertilizer amount and irrigation amount, LAI and SPAD of maize increased gradually, which resulted in the enhancement of light capture ability of maize.

### 4.5 Effects of organic fertilizer application rate and irrigation amount on water consumption and yield WUE of intercropping maize/mung bean

Current research on organic fertilizer-irrigation interactions for crop ET and WUE is predominantly confined to monocropping systems, leaving a critical gap in understanding their effects on intercropping—a practice inherently advantageous for resource-use efficiency. Our findings reveal that while crop water consumption in maize-mung bean intercropping increases with irrigation (I1 and I2 elevated ET by 2.57 ~ 4.29% and 1.70 ~ 3.68% vs. I0, respectively), organic fertilizer application significantly enhances WUE by 8.61 ~ 10.91% in maize and 16.60 ~ 19.74% in mung bean compared to unfertilized controls (**Table 4**). This synergy is theoretically grounded in resource-use complementarity and hydraulic-nutrient interactions, two mechanisms pivotal for optimizing productivity in water-scarce environments.

From a soil-plant hydraulics perspective, organic fertilizers improve soil structure by increasing organic matter content, thereby enhancing water retention and reducing gravitational drainage (Wang et al., 2023; Ning et al., 2024). In intercropping, this creates a “buffer zone” for water availability: maize (a tall, water-demanding crop) and mung bean (a short, drought-tolerant legume) exploit soil water at different depths, while organic matter reduces surface evaporation, directing more water to transpiration—a process directly linked to WUE. This aligns with the “hydraulic lift” hypothesis, where deeper-rooted maize may facilitate water redistribution to shallow-rooted mung bean under moderate irrigation, a phenomenon amplified by improved soil moisture storage from organic amendments (Tolimir et al., 2024).

The nutrient-water interaction effect further explains WUE gains. Organic fertilizers supply slow-release nutrients and bioactive compounds that stimulate root growth and photosynthetic apparatus development (Wang et al., 2021; Zhai et al., 2022; Xu et al., 2024). For instance, enhanced chlorophyll synthesis and stomatal regulation boost carbon assimilation per unit water loss, a mechanism corroborated by our earlier Photosynthetic characteristic data showing higher Pn under fertilized treatments. Critically, in water-limited systems, this nutrient-mediated improvement in “transpiration efficiency” offsets the ET increase from irrigation, creating a net gain in WUE (Sun et al., 2006). This tradeoff is absent in monocropping due to uniform root architecture and resource competition, highlighting intercropping’s advantage in leveraging complementary niche differentiation (Chimonyo et al., 2016).

## 5 Conclusions

This study investigated the effects of organic fertilizer application amount and irrigation amount on leaf growth physiology, photosynthetic characteristics, ET, WUE, dry matter accumulation and yield under maize/mung bean intercropping mode, and the changes of leaf growth physiological characteristics with emergence time. The results showed that the leaf function of maize and mung bean increased first and then decreased with emergence time. This is because when maize silks and mung beans bloom, nitrogen in the leaves is transferred to the seeds to meet the filling needs of the seeds. With the advancement of crop growth period, leaves will gradually age, resulting in a decline in leaf function. The application of organic fertilizer combined with different irrigation amounts can improve the utilization of light energy by maize/mung bean, significantly improve the leaf growth of maize and mung bean and the synthesis and accumulation of chlorophyll, affect the capture and absorption of light by plants, promote the accumulation of photosynthesis, and ensure the stability of yield. In the case of sufficient water, the application of appropriate reduction of organic fertilizer can ensure that the crop does not reduce production and enhance the yield stability of the plant. Secondly, with the increase of irrigation amount, ET of crops also increased correspondingly, and WUE showed a trend of increasing first and then decreasing. The right amount of water can meet the growth needs of crops, promote photosynthesis and dry matter accumulation, so as to improve WUE. However, when the amount of irrigation exceeds a certain limit, excessive water will lead to an increase in ineffective evaporation, which will reduce WUE.

Although this study explored the variation laws of photosynthetic characteristics, evapotranspiration and water use efficiency of crops in the corn-mung bean intercropping model under the coupling of water and fertilizer, as well as the interaction relationships among pn, spad and ab. However, it did not involve individual experiments or experimental designs in different geographical locations and climates. In subsequent studies, we will continue to set up long-term positioning experiments under different conditions for observation. Meanwhile, we are attempting to combine machine learning methods (such as random forest, neural network, etc.) to construct a relationship among water, fertilizer, photosynthetic characteristics and yield. Perhaps due to the influence of the experimental design, the current construction results are not ideal. Specifically, the constructed equation is somewhat monotonous in the setting of water and fertilizer gradients, which cannot be well visualized, and the coupling degree of the output results is insufficient. We will constantly try and explore its internal mechanism through further experiments.

## Data Availability

The original contributions presented in the study are included in the article/supplementary material. Further inquiries can be directed to the corresponding author.
